# Tumor cellularity beyond the visible in soft tissue sarcomas: Results of an ADC-based, single center, and preliminary radiomics study

**DOI:** 10.3389/fonc.2022.879553

**Published:** 2022-10-11

**Authors:** Chiara Giraudo, Giulia Fichera, Paolo Del Fiore, Simone Mocellin, Antonella Brunello, Marco Rastrelli, Roberto Stramare

**Affiliations:** ^1^ Department of Medicine – DIMED, University of Padova, Padova, Italy; ^2^ Soft-Tissue, Peritoneum and Melanoma Surgical Oncology Unit, Veneto Institute of Oncology - IOV Istituto di Ricovero e Cura a Carattere Scientifico (IRCCS), Padova, Italy; ^3^ Department of Surgery, Oncology and Gastroenterology (DISCOG), University of Padua, Padua, Italy; ^4^ Department of Oncology, Medical Oncology 1 Unit, Veneto Institute of Oncology - IOV Istituto di Ricovero e Cura a Carattere Scientifico (IRCCS), Padua, Italy

**Keywords:** radiomics, soft tissue sarcoma, magnetic resonance, tumor grade, metastasis

## Abstract

**Purpose:**

Soft tissue sarcomas represent approximately 1% of all malignancies, and diagnostic radiology plays a significant role in the overall management of this rare group of tumors. Recently, quantitative imaging and, in particular, radiomics demonstrated to provide significant novel information, for instance, in terms of prognosis and grading. The aim of this study was to evaluate the prognostic role of radiomic variables extracted from apparent diffusion coefficient (ADC) maps collected at diagnosis in patients with soft tissue sarcomas in terms of overall survival and metastatic spread as well as to assess the relationship between radiomics and the tumor grade.

**Methods:**

Patients with histologically proven soft tissue sarcomas treated in our tertiary center from 2016 to 2019 who underwent an Magnetic Resonance (MR) scan at diagnosis including diffusion-weighted imaging were included in this retrospective institution review board–approved study. Each primary lesion was segmented using the b50 images; the volumetric region of interest was then applied on the ADC map. A total of 33 radiomic features were extracted, and highly correlating features were selected by factor analysis. In the case of feature/s showing statistically significant results, the diagnostic accuracy was computed. The Spearman correlation coefficient was used to evaluate the relationship between the tumor grade and radiomic features selected by factor analysis. All analyses were performed applying p<0.05 as a significant level.

**Results:**

A total of 36 patients matched the inclusion criteria (15 women; mean age 58.9 ± 15 years old). The most frequent histotype was myxofibrosarcoma (16.6%), and most of the patients were affected by high-grade lesions (77.7%). Seven patients had pulmonary metastases, and, altogether, eight were deceased. Only the feature Imc1 turned out to be a predictor of metastatic spread (p=0.045 after Bonferroni correction) with 76.7% accuracy. The value -0.16 showed 73.3% sensitivity and 71.4% specificity, and patients with metastases showed lower values (mean Imc1 of metastatic patients -0.31). None of the examined variables was a predictor of the overall outcome (p>0.05, each). A moderate statistically significant correlation emerged only between Imc1 and the tumor grade (r=0.457, p=0.005).

**Conclusions:**

In conclusion, the radiomic feature Imc1 acts as a predictor of metastatic spread in patients with soft tissue sarcomas and correlates with the tumor grade.

## Introduction

Soft tissue sarcomas are rare tumors representing approximately 1% of all malignancies, and diagnostic radiology plays a significant role not only at diagnosis and staging but also during follow-up (1–3). In particular, MR imaging, because of its intrinsic soft tissue contrast, is considered the main tool for investigating the primary site. Furthermore, this technique carries additional advantages due to the possibility to perform quantitative and functional analyses. In fact, the information about tumor perfusion, chemical composition, and cellularity can be easily assessed by dynamic contrast-enhanced (DCE) techniques, spectroscopy, and diffusion-weighted imaging (DWI), respectively (3–9). Nowadays, DWI is considered crucial in oncological imaging in general (10, 11). For soft tissue sarcomas, it has been demonstrated that it also contributes in distinguishing recurrences from postsurgical scars, improves specificity in defining tumor margin infiltration, and predicts the response to treatment (8–10). Despite these encouraging results, potential pitfalls and controversies must be addressed, especially for myxoid tumors due to their high mucin content. In fact, in this case, the distinction between benign and malignant lesions by DWI could be hampered (10).

The recent technical developments allowed radiologists to move further in their contribution to the overall diagnostic management of cancer patients as demonstrated by the increasing use of complex analyses such as histogram and radiomics. Indeed, these types of computations were demonstrated to be very useful for different types of tumors including soft tissue sarcomas, applying various sequences (12–15). For instance, Corino et al. already showed that the variables of first order (FOS) extracted from apparent diffusion coefficient (ADC) maps allow the distinction of the lesions of different grades. Similar conclusions were drawn by Xu and colleagues using T1w and T2w fat-sat imaging (15, 16). Using DCE-MRI at the baseline in patients with high-grade non-metastatic soft tissue sarcomas, Crombe et al. showed that radiomic variables have an important prognostic role (17). Lastly, Gao and colleagues did not predict the response to radiotherapy by DWI but better predicted the treatment effect score applying delta radiomics (18).

Despite this growing evidence, up to now, to the best of our knowledge, the prognostic value of radiomics regarding metastatic spread has not been fully investigated yet.

Thus, the aim of this study was to evaluate the prognostic role of radiomic variables extracted from ADC maps collected at diagnosis in patients with soft tissue sarcomas in terms of overall survival and metastatic spread as well as to assess the relationship between radiomics and the tumor grade.

## Methods

**Figure 1:**
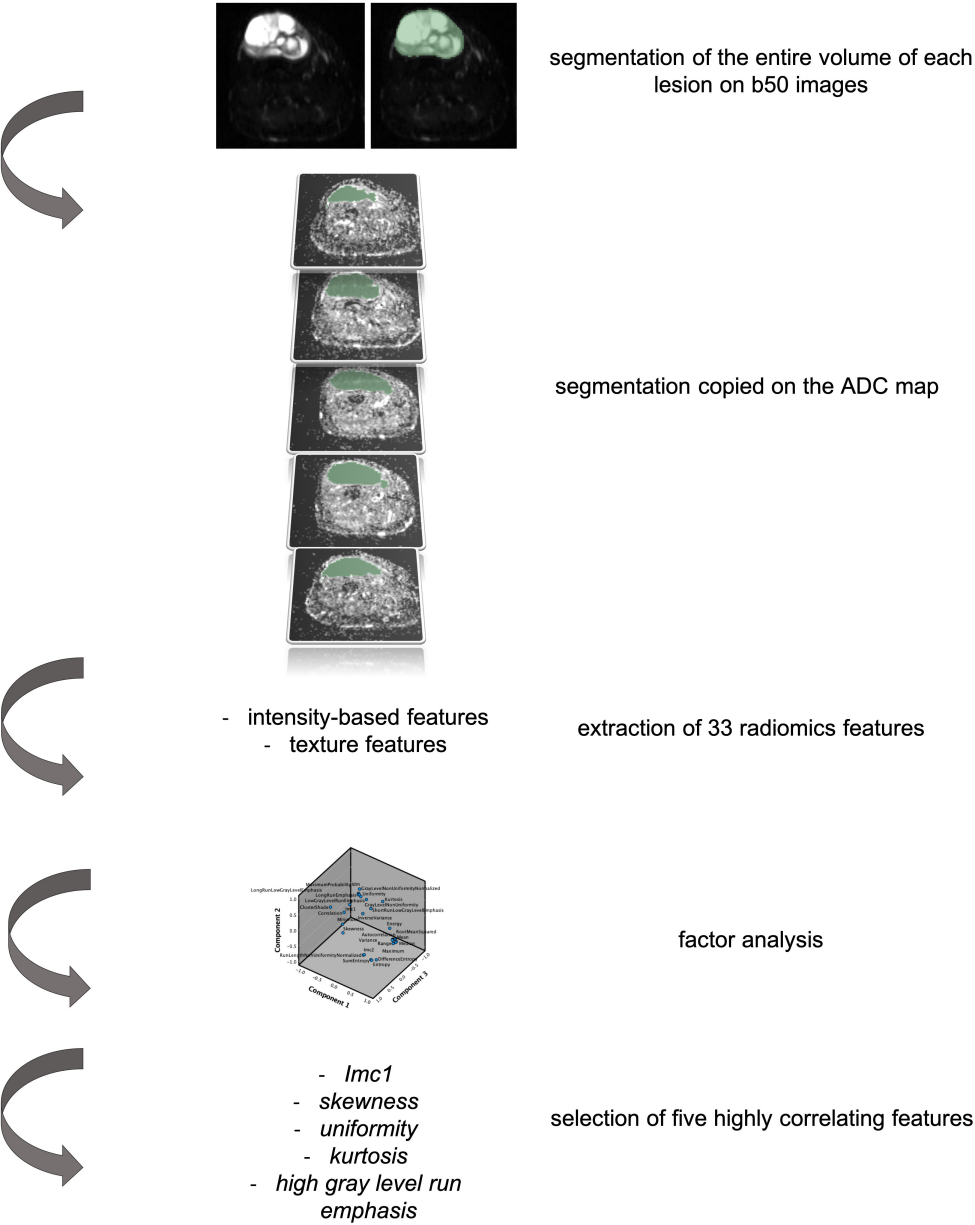
Representation of the segmentation process and data extraction from the primary lesion of patients with soft tissue sarcoma included in the study. In particular, it is hereby represented by a 76-year-old woman affected by a myxofibrosarcoma of the right lower leg. The axial b50 DWI images have been used for segmentation of the entire lesion. The volumetric segmentation has been then applied on the apparent diffusion coefficient (ADC) map and the extraction of 33 radiomic features performed. Factor analysis allowed the selection of five highly correlating features.

Patients with histologically proven soft tissue sarcomas treated in our tertiary center from 2016 to 2019 who underwent an MR scan at diagnosis by a 1.5 T scanner (Siemens Avanto 1.5T, Siemens Healthcare, Siemens, Erlangen, Germany) including an axial short tau inversion recovery–DWI sequence with 6 mm slice thickness and two b-values (i.e., b50 and b 800) were examined for this preliminary retrospective single-center institution review board–approved study. One radiologist with 12 years of experience in musculoskeletal imaging segmented each lesion along tumor margins using the b50 images because of the higher spatial resolution than the ADC map. The volumetric region of interest was then applied on the map (**Figure 1**). From each segmented volume, the 33 radiomic features of two classes were extracted: intensity-based features (FOS) and texture features [gray-level co-occurrence matrix (GLCM as well as gray-level run length matrix (GLRLM)]. The segmentation and extraction of radiomics features have been performed by an open-source software (3D Slicer, www.slicer.org).

### Statistical analysis

Descriptive statistics were applied for demographics, tumor histotype, site, grade, and metastatic spread. Factor analysis was applied to select highly correlating radiomic features. Then, stepwise regression analysis was used to evaluate if any of the selected radiomic variables had a predicting role on the overall outcome (dead/alive) and/or the metastatic spread. Moreover, the Bonferroni correction was applied to correct the statistical significance level for multiple tests. In the case of feature/s showing statistically significant results, the diagnostic accuracy was computed using ROC curves and the value/s with the highest Youden index were selected as a cut-off. The Spearman correlation coefficient was used to evaluate the relationship between the tumor grade and all radiomic features selected by factor analysis.

To evaluate the robustness of the proposed method, all segmentations and data extraction were repeated by a second reader with 4 years of experience in oncological imaging and the intraclass correlation coefficient (ICC) of the variables highly correlating at factor analysis were computed. ICC values >.750 were considered excellent (19).

All statistical analyses were performed with SPSS (IBM SPSS Statistics version 27, IBM Armonk, NY, USA), applying p<0.05 as a significant level.

## Results

From an overall amount of 80 cases treated in our center, 36 patients matched the inclusion criteria (15 women; mean age 58.9 ± 15 years old) and were examined. The characteristics of the examined population are summarized in **Table 1**. The most frequent histotype was myxofibrosarcoma (six patients, 16.6%), and most of the patients were affected by high-grade lesions (i.e., 28 had grade III lesions, 77.7%). Seven patients had pulmonary metastases, and, altogether, eight were deceased. On average, the survival was of 56.9 ± 22 months. Most of the lesions affected the lower limbs (29, 80.5%).

**Table 1:** Characteristics of the examined population.

**Gender**(female/male)	14/22
**Age**(years)	58.9 ± 15 (mean ± SD)(range 18–82)
**Histotype**	6 myxofibrosarcoma
	6 undifferentiated pleomorphic sarcoma
	5 leiomyosarcoma
	4 myxoid liposarcoma
	3 aggressive fibromatosis
	2 synovial sarcoma
	2 solitary fibrous tumor
	2 liposarcoma
	2 round cell liposarcoma
	1 angiosarcoma
	1 hemangioendothelioma
	1 malignant peripheral nerve sheath tumor
	1 spindle cell sarcoma
**Site**	29 lower limbs
	3 upper limbs
	2 pelvis
	2 thorax
	1 neck
**Grade**	3 grade I
	5 grade II
	28 grade III
**Metastatic**(yes/no)	7/29
**Deceased**(yes/no)	8/28
**Survival in months**(median)	55

Factor analysis allowed the extraction of five highly correlating variables: three of first order (kurtosis, skewness, and uniformity) and two of second order (informational measure of correlation (Imc1) and high gray-level run emphasis).

**Figure 2:**
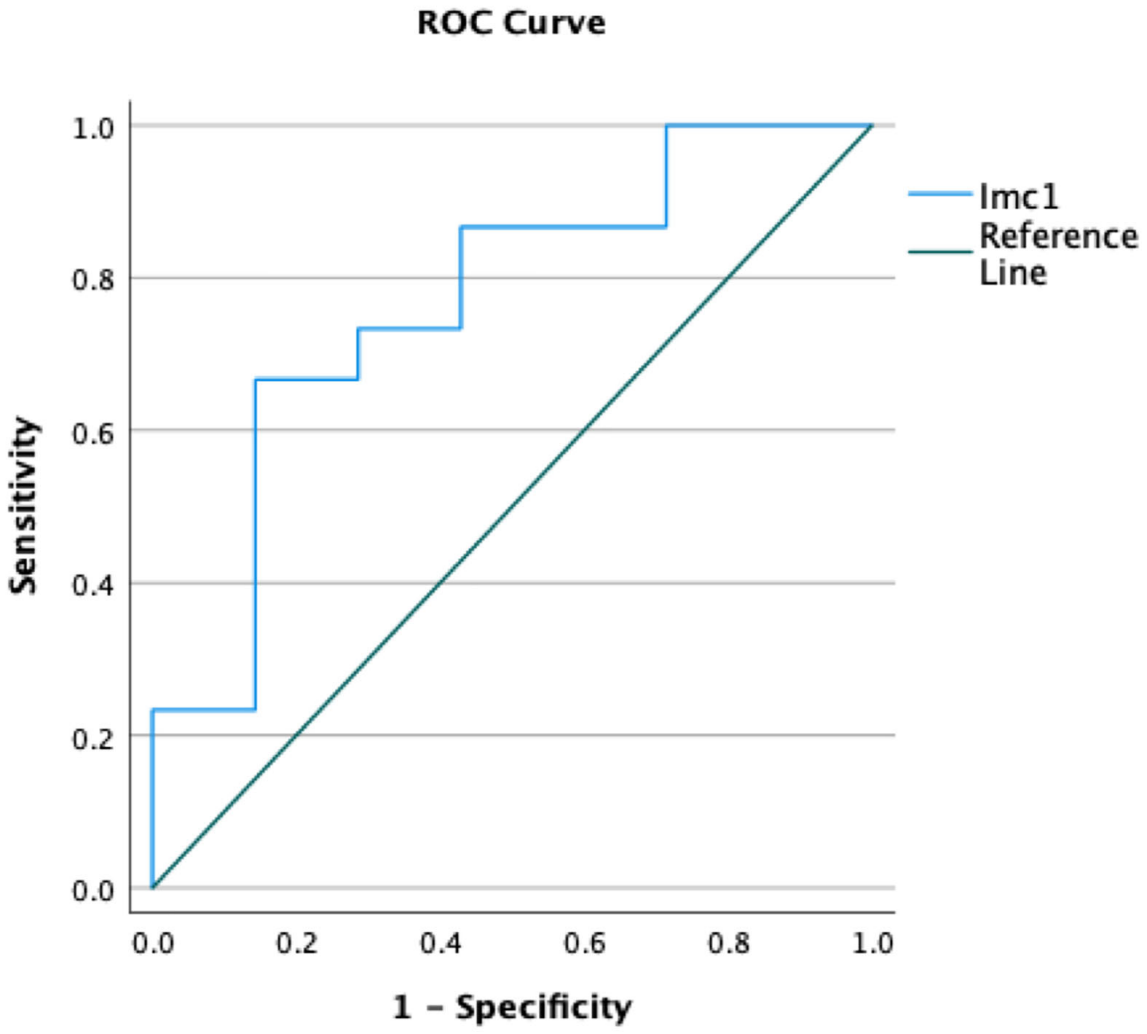
Receiver operating curves (ROCs) demonstrating the accuracy of the radiomic feature Imc1 in predicting the occurrence of metastases in patients with soft tissue sarcomas.

Only the feature Imc1 turned out to be a predictor of metastatic spread (p=0.045 after Bonferroni correction) with 76.7% accuracy (**Figure 2**). The value -0.16 showed 73.3% sensitivity and 71.4% specificity, and patients with metastases showed lower values (mean Imc1 of metastatic patients -0.31). None of the examined variables was a predictor of the overall outcome (p>0.05 each).

**Figure 3:**
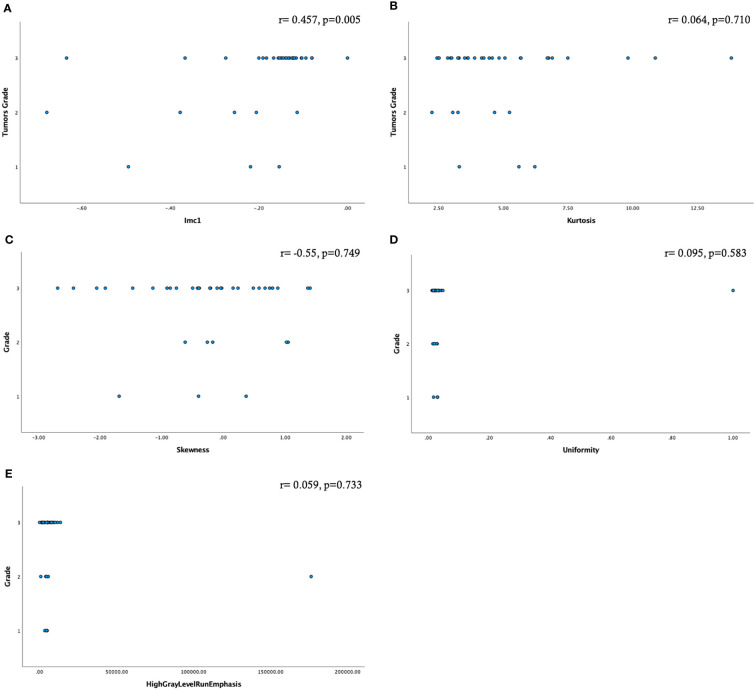
Scatter plots showing the relationship among the radiomic features selected by factor analysis and the tumor grade **(A–E)**. Only the feature Imc1 showed a moderate statistically significant correlation **(A)**.

A moderate statistically significant correlation emerged only between Imc1 and the tumor grade (r=0.457, p=0.005) (**Figure 3**).

The proposed method of the segmentation and extraction of radiomic features showed high reproducibility: kurtosis ICC = .870 [95% CI,.748 –.933], skewness ICC = .765 [95% CI,.544 –.879], uniformity ICC = .814 [95% CI,.640 –.904], Imc1 ICC = .786 [95%CI,.584–.890], and high gray-level run emphasis ICC=.842 [95% CI,.692 –.918].

## Discussion

To the best of our knowledge, this is one of the largest studies investigating the role of radiomic features extracted from the ADC maps of patients with soft tissue sarcomas at diagnosis demonstrating that the Imc1 is a predictor of metastatic spread and correlates with the tumor grade. Similar results were obtained using T1w and T2w fat-sat sequences by other groups. For instance, Tian and colleagues, applying a machine learning model, and Vallieres et al., associating the above-mentioned MR sequences with positron emission tomography (PET) information, obtained an early detection of pulmonary metastases (13, 20). This last evidence, together with other previous promising results provided by PET/MR-based histogram analyses and texture features, suggests that the role of hybrid imaging should be further assessed, especially about the possibility to simultaneously collect data regarding metabolic activity and functional information (12, 20).

Regarding, in particular, the Imc1 feature, it quantifies the complexity of the texture and its importance has been previously demonstrated in several studies on different types of cancer (21–24). For instance, our results are in line with those of Liao and colleagues who identified more negative values in patients with brain metastasis due to non-small lung cancer with poor local tumor control, thus suggesting that tumors with high intralesion heterogeneity might be associated with a worse clinical course, in our study represented by the occurrence of metastasis. Moreover, together with other features, it was part of a “radiomic signature” indicating patients with ground glass nodules at risk for invasive adenocarcinoma (25). The feature Imc1 may even be considered a novel biomarker of metastatic spread in patients with soft tissue sarcomas considering that it showed an excellent repeatability in our dataset and strong robustness in a previously published computed tomography–based computational model (26). Nevertheless, it should be considered that further studies on a larger population are needed to fully assess the hereby presented evidence; since, for instance, Corino et al., in a group of 19 patients with soft tissue sarcomas, showed that only the features of the first order were the best classifier of the tumor grade (15).

For the overall role of radiomics for this heterogeneous group of tumors, as previously underlined, different MR sequences have been used with very promising results (17, 18) and probably not only more comparisons among sequences are necessary (27), but it should also be assessed if a model combining information deriving from multiple sequences could provide additional and more robust results.

In contrast to part of the literature, in our study, none of the selected radiomic features turned out to be a predictor of the overall outcome. This discrepancy might be due to the heterogeneity of our sample in terms of the histotype and grade. In fact, radiomics acted as prognostic factor in selected groups like patients with myxoid and liposarcomas only and patients with high-grade lesions (28–30). Moreover, in the above-mentioned studies, other types of sequences (i.e., T1w, T2 fat-sat, or DCE-MRI) than DWI have also been used. We strongly encourage further investigation in this direction to also gain new knowledge about the potential prognostic role of this sequence in the specific subgroups of patients.

This study is affected by several limits. First, as noted above, the sample size and the large variety of histotypes did not allow to investigate the predictive role of this technique on the type of tumor and the relationship between different histotypes and radiomic features. Moreover, the small population also hampered the subdivision of our sample in test and validation sets. Certainly, the rarity of the disease contributed to these flaws and multicenter studies are necessary to fill these voids and provide further insights into the role of radiomics for these tumors.

Then, the lower spatial resolution of DWI in contrast to conventional imaging should be considered. Nevertheless, as previously mentioned, analyses showed a high repeatability and several *ex vivo* and *in vivo* studies demonstrated a high reliability of radiomics using this type of sequence (31, 32).

In conclusion, the radiomic feature Imc1 acts as a predictor of metastatic spread in patients with soft tissue sarcomas and correlates with the tumor grade. Further studies on a larger sample and including delta-radiomics analyses at follow-up are expected to provide new insights on the potential impact of this evidence on the therapeutic and overall management of this rare group of tumors.

## Data availability statement

The raw data supporting the conclusions of this article will be made available by the authors, without undue reservation.

## Ethics statement

This study was reviewed and approved by the Ethics Committee of the Veneto Institute of Oncology (Approval No. 16336/21 CESC-IOV) on 1 September 2021.

## Author contributions

All authors significantly contributed to this study and approved the final version of the manuscript. GC: study design, data analysis, and manuscript drafting. FG: data collection and review and editing of the manuscript. DP: data collection and review and editing of the manuscript. MS: data interpretation and review and editing of the manuscript. BA: assisted in data collection and review and editing of the manuscript. RM: study design and review and editing of the manuscript. SR: review and editing of the manuscript and study coordination. All authors contributed to the article and approved the submitted version.

## Conflict of interest

The authors declare that the research was conducted in the absence of any commercial or financial relationships that could be construed as a potential conflict of interest.

## Publisher’s note

All claims expressed in this article are solely those of the authors and do not necessarily represent those of their affiliated organizations, or those of the publisher, the editors and the reviewers. Any product that may be evaluated in this article, or claim that may be made by its manufacturer, is not guaranteed or endorsed by the publisher.

## References

[1] Von Mehren M Randall LR Benjamin RS Boles S Bui MM Ganjoo KN. Soft tissue sarcoma, version 2. 2018. NCCN clinical practice guidelines in oncology. J Natl Compr Canc Netw (2018) 16:5. doi: 10.6004/jnccn.2018.0025 29752328

[2] Robinson E Bleakney RR Ferguson PC O’Sullivan B. Oncodiagnosis panel: 2007. multidisciplinary management of soft-tissue sarcoma. RadioGraphics (2008) 28:7. doi: 10.1148/rg.287085167 19001660

[3] Varma DGK. Imaging of soft-tissue sarcomas. Curr Oncol Rep (2000) 2:6. doi: 10.1007/s11912-000-0100-2 11122882

[4] Subhawong TK Jacobs MA Fayad LM. Diffusion-weighted MR imaging for characterizing musculoskeletal lesions. RadioGraphics (2014) 34:5. doi: 10.1148/rg.345140190 PMC4319521 25208274

[5] Li X Wang Q Dou Y Zhang Y Tao J Yang L. Soft tissue sarcoma: can dynamic contrast-enhanced (DCE) MRI be used to predict the histological grade? Skeletal Radiol (2020) 49:11. doi: 10.1007/s00256-020-03491-z 32519183

[6] Lee JH Yoon YC Seo SW Choi YL Kim HS. Soft tissue sarcoma: DWI and DCE-MRI parameters correlate with ki-67 labeling index. Eur Radiol (2020) 30:2. doi: 10.1007/s00330-019-06445-9 31630234

[7] Subhawong TK Wang X Durand DJ Jacobs MA Carrino JA Machado AJ. Proton MR spectroscopy in metabolic assessment of musculoskeletal lesions. AJR (2012) 198:1. doi: 10.2214/AJR.11.6505 22194493 PMC4868063

[8] Del Grande F Subhawong TK Weber K Aro M Mugera C Fayad LM. Detection of soft-tissue sarcoma recurrence: Added value of functional MR imaging techniques at 3.0 T. Radiology (2014) 271:2. doi: 10.1148/radiol.13130844 24495264

[9] Soldatos T Ahlawat S Montgomery E Chalian M Jacobs MA Fayad LM. Multiparametric assessment of treatment response in high-grade soft-tissue sarcomas with anatomic and functional MR imaging sequences. Radiology (2016) 278:3. doi: 10.1148/radiol.2015142463 PMC4770945 26390048

[10] Messina C Bignone R Bruno A Bruno A Bruno F Calandri M. Diffusion-weighted imaging in oncology: An update. Cancers (2020) 12:6. doi: 10.3390/cancers12061493 PMC7352852 32521645

[11] Giraudo C Karanikas G Weber M Raderer M Jager U Simonitsch-Klupp I. Correlation between glycolytic activity on [18F]-FDG-PET and cell density on diffusion-weighted MRI in lymphoma at staging. J Magn Reson Imaging (2018) 47:5. doi: 10.1002/jmri.25884 29086453

[12] Orsatti G Zucchetta P Varotto A Crimì F Weber M Cecchin D. Volumetric histograms-based analysis of apparent diffusion coefficients and standard uptake values for the assessment of pediatric sarcoma at staging: preliminary results of a PET/MRI study. Radiol Med (2021) 126:6. doi: 10.1007/s11547-021-01340-0 33683542

[13] Tian L Zhang D Bao S Nie P Hao D Liu Y. Radiomics-based machine-learning method for prediction of distant metastasis from soft-tissue sarcomas. Clin Radiol (2021) 76:2. doi: 10.1016/j.crad.2020.08.038 33293024

[14] Tagliafico AS Bignotti B Rossi F Valdora F Martinoli C. Local recurrence of soft tissue sarcoma: a radiomic analysis. Radiol Oncol (2019) 53:3. doi: 10.2478/raon-2019-0041 PMC6765164 31553702

[15] Corino VDA Montin E Messina A Casali PG Gronchi A Marchianò A. Radiomic analysis of soft tissues sarcomas can distinguish intermediate from high-grade lesions. J Magn Reson Imaging (2018) 47:3. doi: 10.1002/jmri.25791 28653477

[16] Xu W Hao D Hou F Zhang D Wang H. Soft tissue sarcoma: Preoperative MRI-based radiomics and machine learning may be accurate predictors of histopathologic grade. AJR (2020) 215:4. doi: 10.2214/AJR.19.22147 32755226

[17] Crombe A Saut O Guigui J Italiano A Buy X Kind M. Influence of temporal parameters of DCE-MRI on the quantification of heterogeneity in tumor vascularization. J Magn Reson Imaging (2019) 50:6. doi: 10.1002/jmri.26753 30980697

[18] Gao Y Kalbasi A Hsu W Ruan D Fu J Shao J. Treatment effect prediction for sarcoma patients treated with preoperative radiotherapy using radiomics features from longitudinal diffusion- weighted MRI. Phys Med Biol (2020) 65:17. doi: 10.1088/1361-6560/ab9e58 32554891

[19] Cicchetti DV. Guidelines, criteria, and rules of thumb for evaluating normed and standardized assessment instruments in psychology. Psychol Assess (1994) 6:4. doi: 10.1037/1040-3590.6.4.284

[20] Vallieres M Freeman CR Skamene SR Naqa IE. A radiomics model from joint FDG-PET and MRI texture features for the prediction of lung metastases in soft-tissue sarcomas of the extremities. Phys Med Biol (2015) 60:14. doi: 10.1088/0031-9155/60/14/5471 26119045

[21] Tietz E Truhn D Mueller-Franzes G Berres ML Hamesch K Lang SA. A radiomics approach to predict the emergence of new hepatocellular carcinoma in computed tomography for high-risk patients with liver cirrhosis. Diagnostics (2021) 11:9. doi: 10.3390/diagnostics11091650 PMC8471809 34573991

[22] Liao CY Lee CC Yang HC Chen CJ Chung WY Wu HS. Enhancement of radiosurgical treatment outcome prediction using MRI radiomics in patients with non-small cell lung cancer brain metastases. Cancers (2021) 13:16. doi: 10.3390/cancers13164030 PMC8392266 34439186

[23] Wang X Wan Q Chen H Li Y Li X. Classification of pulmonary lesion based on multiparametric MRI: utility of radiomics and comparison of machine learning methods. Eur Radiol (2020) 30:8. doi: 10.1007/s00330-020-06768-y 32222795

[24] Shin J Lin JS Huh YM Kim JH Hyung WJ Chung JJ. A radiomics-based model for predicting prognosis of locally advanced gastric cancer in the preoperative setting. Sci Rep (2021) 11:1. doi: 10.1038/s41598-021-81408-z 33479398 PMC7820605

[25] Jiang Y Che S Ma S Liu X Guo Y Liu A. Radiomic signature based on CT imaging to distinguish invasive adenocarcinoma from minimally invasive adenocarcinoma in pure ground-glass nodules with pleural contact. Cancer Imaging (2021) 21:1. doi: 10.1186/s40644-020-00376-1 33407884 PMC7788838

[26] Bernatowicz K Grussu F Ligero M Garcia A Delgado E Perez-Lopez R. Robust imaging habitat computation using voxel-wise radiomics features. Sci Rep (2021) 11:1. doi: 10.1038/s41598-021-99701-2 34635786 PMC8505612

[27] Hu P Chen L Zhou Z. Machine learning in the differentiation of soft tissue neoplasms: Comparison of fat-suppressed T2WI and apparent diffusion coefficient (ADC) features-based models. J Digital Imaging (2021) 34:5. doi: 10.1007/s10278-021-00513-7 PMC8554992 34545474

[28] Crombe A Le Loarer F Sitbon M Italiano A Stoeckle E Buy X. Can radiomics improve the prediction of metastatic relapse of myxoid/round cell liposarcomas? Eur Radiol (2020) 30:5. doi: 10.1007/s00330-019-06562-5 31953663

[29] Crombe A Fadli D Buy X Italiano A Saut O Kind M. High-grade soft-tissue sarcomas: Can optimizing dynamic contrast-enhanced MRI postprocessing improve prognostic radiomics models? J Magn Reson Imaging (2020) 52:1. doi: 10.1002/jmri.27040 31922323

[30] Crombe A Perier C Kind M De Senneville BD Le Loarer F Italiano A. T2-based MRI delta-radiomics improve response prediction in soft-tissue sarcomas treated by neoadjuvant chemotherapy. J Magn Reson Imaging (2019) 50:2. doi: 10.1002/jmri.26589 30569552

[31] Dreher C Kuder TA Koenig F Mlynarska-Bujny A Tenconi C Paech D. Radiomics in diffusion data: a test-retest, inter- and intra-reader DWI phantom study. Clin Radiol (2020) 75:10. doi: 10.1016/j.crad.2020.06.024 32723501

[32] Peerlings J Woodruff HC Winfield JM Ibrahim A Van Beers BB Heerschap A. Stability of radiomics features in apparent diffusion coefficient maps from a multi-centre test-retest trial. Sci Rep (2019) 9:1. doi: 10.1038/s41598-019-41344-5 30886309 PMC6423042

